# Molecular Signatures of Exercise Adaptation in Arabian Racing Horses: Transcriptomic Insights from Blood and Muscle

**DOI:** 10.3390/genes16040431

**Published:** 2025-04-04

**Authors:** Monika Stefaniuk-Szmukier, Tomasz Szmatoła, Katarzyna Ropka-Molik

**Affiliations:** 1 Department of Animal Molecular Biology, National Research Institute of Animal Production, Krakowska 1, 32-083 Balice, Poland; katarzyna.ropka@iz.edu.pl; 2 Center for Experimental and Innovative Medicine, The University of Agriculture in Krakow, Rędzina 1c, 30-248 Kraków, Poland; tomasz.szmatola@iz.edu.pl

**Keywords:** Arabian horse, endurance racing, exercise adaptation, RNA sequencing, transcriptomics, differentially expressed genes, muscle metabolism, equine sports genetics

## Abstract

**Background/Objectives:** Human-driven selection has shaped modern horse breeds into highly specialized athletes, particularly in racing. Arabian horses, renowned for their endurance, provide an excellent model for studying molecular adaptations to exercise. This study aimed to identify genes commonly influenced by physical exertion in the gluteus medius muscle and whole blood of Arabian horses during their first year of race training. **Methods:** RNA sequencing of sixteen pure-breed Arabian horses was used to analyze transcriptomic changes at three key training stages. Differentially expressed genes (DEGs) were identified to explore their role in endurance and metabolic adaptation. **Results:** Seven genes—*RCHY1*, *PIH1D1*, *IVD*, *FABP3*, *ANKRD2*, *USP13*, and *CRYAB*—were consistently deregulated across tissues and training periods. These genes are involved in muscle remodeling, metabolism, oxidative stress response, and protein turnover. *ANKRD2* was associated with mechanosensing and muscle adaptation, *FABP3* with fatty acid metabolism, and *USP13* with ubiquitination-related pathways crucial for muscle recovery and energy regulation. The transcriptomic overlap between muscle and blood suggests potential systemic biomarkers for athletic performance and endurance. **Conclusions:** Our findings highlight the importance of multi-tissue transcriptomic profiling in understanding exercise-induced molecular adaptations. The identified genes warrant further investigation as potential molecular markers for monitoring training progression and athletic potential in endurance horses. This study contributes to the growing field of equine sports genetics and may offer translational insights into human sports performance.

## 1. Introduction

The genetic makeup of modern horses has been shaped by human needs. Since domestication, strong selection processes have led to the development of animals specialized for various tasks [1]. Over centuries, horses have played crucial roles in transportation, warfare, and food production, contributing significantly to the advancement of civilization [2]. As evolutionary adaptations of running grazers merged with human-driven selection, modern horses became highly specialized athletes. Their unique physiological traits enable them to endure extreme physical exertion, making them valuable models for human sports science, regenerative medicine, and studies on molecular responses to exercise.

Equestrian racing was part of the Ancient Olympic Games, which began in 776 BC, though the origins of the first horse race remain unknown. Over time, racing became known as the “sport of kings” evolving into a highly lucrative global industry generating billions in revenue annually. Various horse breeds participate in racing, including Thoroughbreds [3], Arabians [4], Quarter Horses [5], and Norwegian–Swedish Coldblooded Trotters [6]. Among these, Thoroughbreds are bred exclusively for flat racing, leading to the early identification of genetic loci associated with racing performance [7,8].

Recent research has utilized advanced techniques such as transcriptomics, miRNAomics, and metabolomics to explore the genetic basis of racing and endurance traits in Arabians [9,10,11,12].

Genome-wide studies have identified genetic variants associated with exercise performance, such as *SORCS3*, *SLC39A12*, and *KCNQ1OT1*, which have been linked to endurance racing performance and cardiac rhythm regulation [13]. Additionally, *SLC16A1*, a gene involved in lactate metabolism, has been associated with adaptation to high-intensity exercise, while selection signatures in *COX4I1*, *ADCY1*, *GAD1*, and *CBLB* suggest their roles in ATP synthesis, vascular function, and oxidative metabolism [14,15] Transcriptomic analyses have revealed key genes involved in metabolic adaptation, muscle remodeling, and stress response. Genes related to fatty acid degradation (*ACAA2*, *ACADS*, *ACADM*, *ACSL1*, *CPT1B*, and *CPT2*) are highly expressed in Arabian horses undergoing endurance training, emphasizing their role in energy metabolism [15]. Additionally, long-term exercise induces immune and inflammatory responses, as evidenced by the upregulation of *IL6ST*, *IL6R*, *IL7R*, and *ITGA4*. Muscle remodeling processes are further supported by changes in *SH3RF2*, which regulates apoptosis and tissue regeneration [16]. A panel of genes, including *PK3CG*, *FOXO3*, *ME3*, *ACTN3*, *PPARA*, *TPM3*, *TNNC1*, *TNNI3*, *TGFBR1*, *TGFBR2*, and *FABP3*, has also been identified as potentially influencing racing performance in Arabian horses. Additionally, miRNA expression studies highlight their role in regulating physiological adaptation to exercise. miR-21-5p, miR-181b-5p, and miR-505-5p have been strongly associated with endurance adaptation, while transcription factors such as *ZFP42*, *SPI1*, *FOXO3*, *IRF3*, and *NRF1* modulate oxidative stress and metabolic pathways through miRNA interactions [10]. Further studies have identified let-7b-5p, miR-16-5p, miR-92a-3p, and miR-192-5p as key regulators of metabolic adaptation in response to endurance exercise [17].

Arabian horses are often regarded as an ancient breed [18], and are subject to selective pressures for different traits, depending on their intended purpose. French Arabians are bred for racing, straight Egyptian Arabians for head and neck conformation, U.S. Arabians for an idealized type, and others for endurance performance. This selective breeding has resulted in considerable variation within the breed, creating highly specialized subpopulations recognized within the Arabian horse lineage [19].

The Arabian breed is also renowned for its exceptional stamina. It has been demonstrated that Arabian horses have higher free fatty acid concentration and utilization in their blood compared with Thoroughbred horses during exercise, which contributes to their endurance capabilities [20]. However, within the breed, different populations have been shaped by distinct selection pressures. For example, French purebred Arabian horses specialize in flat racing, much like Thoroughbreds, while the Polish population follows a more balanced approach to selection and breeding, maintaining both racing performance and endurance capabilities for long-distance riding.

In this study, we aimed to identify genes commonly influenced by exercise in the gluteus medius muscle and whole blood of racing Arabian horses during their first year of training. These genes may serve as potential predictors of fitness, warranting further evaluation.

## 2. Materials and Methods

### 2.1. Ethic Statement

The protocol was approved by the Animal Care and Use Committee of the Institute of Pharmacology, Polish Academy of Sciences in Cracow (no. 1173/2015). High-throughput sequencing (HTS) techniques, particularly RNA-seq, are increasingly used to study gene expression. Although current RNA-seq experiments often involve few biological replicates due to high costs, recently decreasing costs will likely lead to additional follow-up studies, necessitating methods to jointly analyze data from multiple experiments.

### 2.2. Animals and Study Design

The study sample included a group of 16 pure-breed Arabian horses (n = 16; n = 11 males, n = 5 females). The horses involved in all parts of the study were purebred Arabians, belonging to bloodlines with documented origins of more than 200 years. The pedigree study excluded individuals with pedigree foreign lines, introduced to industry just before 1996. All horses were registered in the PASB (Polish Arabian Stood Book). Individuals were owned, bred, and trained by one authority, raised on the same farm, and during all training, competing, and sampling were fed in comparable ways. Exercise and training protocols of the sampled horses were as previously described [15,21]. For this study, the following samples were selected: Whole blood (b) and muscle (m) were collected 3 times. The first samples (Ab and Am) were collected in autumn, the year before the horses were to compete at the race track, when horses were 2.5 years old and had been selected for flat racing training. The second samples (Bb and Bm) were collected after 12 weeks of initial light training, during which the horses increased their trotting intensity. The third (Cb and Cm) collection was conducted after the racing season, once the horses had reached sufficient fitness and successfully competed on the race track. The training schedule was as follows: At 2.5 years of age, the horses entered the training center in autumn and began a structured program to prepare for flat racing, progressing through consecutive training phases as they built the necessary fitness. During the first month (October), they were trained to be ridden (first biopsy; untrained horses). The next phase focused on conditioning, incorporating slow cantering interspersed with walking or trotting (March). As training progressed, the canter distance was gradually increased to 5000 m over the course of a month, followed by a reduction to 2500–3000 m. In the final stage, spanning the seventh month, the gallop distance was progressively extended from 200 m to 1200 m each week. A few days before the racing season commenced, each horse completed a gallop of no more than 2000 m and no less than 200–400 m beyond the planned race distance (second biopsy; horses before the racing season). The average number of starts per horse in the racing season was 6.

### 2.3. Acquisition of Biological Samples and RNA Seq Pre-Processing

The whole blood, biopsy sampling, and RNA sequencing protocols were previously described [15,16]. Briefly, the whole-blood samples (n = 16) were collected from the jugular vein into a Tempus™ Blood RNA Tube (Thermo Scientific, Waltham, MA, USA). The muscle samples (n = 16) collected from gluteus medius muscle were harvested by a ProMag™ Ultra Automatic Biopsy Instrument (Argon Medical Devices Inc., Plano, TX, USA). The RNA from whole blood was isolated by using the MagMAX™-96 Total RNA Isolation Kit (Thermo Scientific, Waltham, MA, USA), according to the attached protocol. For muscle samples, the method described by Chomczyński [22] with the use of Tri Reagent (Thermo Scientific, Waltham, MA, USA) was performed. The quality and quantity of obtained RNA were estimated on a TapeStation 2200 (Agilent Technologies, Santa Clara, CA, USA) by using RNA Screen Tape and an RNA Screen Tape Ladder. The RNA samples with RIN (RNA integrity number) above 8.5 were used for further investigations. Additionally, RNA was visualized by 2% agarose gel electrophoresis to determine its degradation degree. A 400 ng sample of total RNA was used to prepare cDNA libraries with the use of the TruSeq RNA Kit v2 (Illumina, San Diego, CA, USA), according to the manufacturer’s instructions. The quality and quantity of obtained libraries were evaluated by a Qubit 2.0 (Thermo Scientific, Waltham, MA, USA, Invitrogen, Life Technologies) and a TapeStation 2200. The 75 bp paired-end run was performed on the HiScanSQ platform (Illumina, San Diego, CA, USA) using the TruSeq SR Cluster Kit v3- CBOT-HS and TruSeq SBS Kit v3-HS chemistry (Illumina, San Diego, CA, USA). The quality control of acquired sequences was performed by FastQC software (v0.11.5), then filtered to retain reads longer than 36 bp and with a quality score higher than 20 by Flexbar [23]. The raw data of samples were deposited in functional genomics database—Gene Expression Omnibus (GEO)—and assigned GEO accession numbers: GSE88951 (m) and GSE83404 (b). They were then submitted for bioinformatics analysis.

Gene and transcript quantification was performed by using RSEM and STAR aligner and reads were mapped to the Equus Caballus genome (assembly EquCab3). For the detection of differentially expressed genes (DEGs), the R-project package DESeq2 was used [24]. The normalized expression levels were compared between groups A_B, B_C, and A_C for whole blood (b) and muscles (m) and significant differences were considered by adjusted *p* values ≤ 0.1. Adjustment to the *p* values was made to account for multiple testing with the false discovery rate (FDR) method of Benjamini and Hochberg [25].

## 3. Results

### Identification and Analysis of DEGs

To identify transcripts differentially expressed in response to varying levels of physical effort associated with racing performance, we compared the transcriptome data of the two tissues: the whole blood (b) and muscle (m), across three sampling points for both muscle and blood: A_B vs. B_C vs. A_C. The results identified sets of differentially expressed DEGs in both tissues.

**Figure 1:**
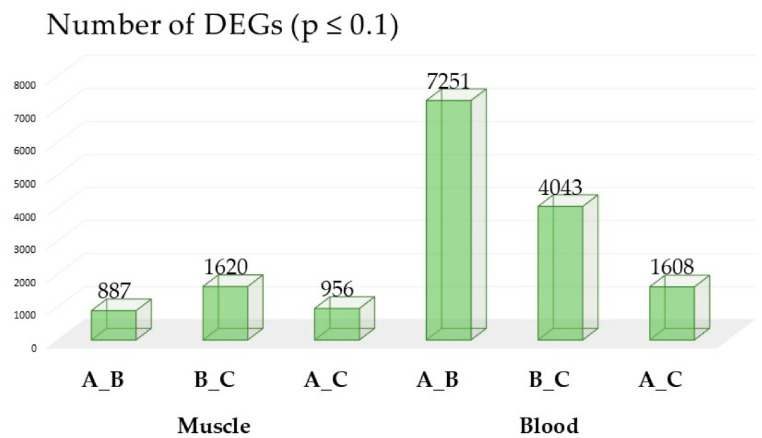
The number of differentially expressed genes (DEGs) (*p* ≤ 0.1) identified in muscle and blood at different stages of training. The highest number of significant DEGs was observed in blood, with 7251 DEGs at the initial stage of training (A_B), followed by a decrease in subsequent stages (B_C: 4043 and A_C: 1608). In muscle tissue, the number of DEGs was lower overall, with 887 DEGs at the initial stage (A_B) and an increase during the second stage (B_C: 1620), before decreasing again (A_C: 956).

After corrections for multiple testing, the highest number of significantly differentiated DEGs were identified in blood. In the initial period of training (A_B) the number of DEGs was 7251 and this decreased at each stage of training (B_C: 4043 and A_C: 1608). Results for muscle tissue showed relatively lower number of DEGs, with the lowest number at initial period of training (A_B: 887) and with the highest number at second period of training (B_C: 1620) (Figure 1).

**Figure 2:**
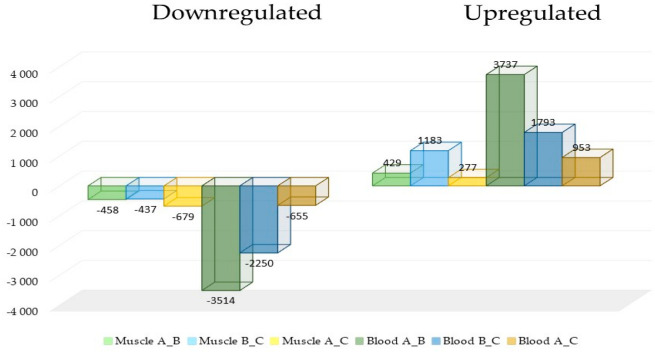
The comparison of differentially expressed genes (DEGs) in muscle and blood tissues revealed variations in gene expression across different training stages. While both upregulated and downregulated genes were observed, the blood samples exhibited significantly greater numbers of DEGs compared with muscle tissue.

The comparison of DEGs in both tissues identified increases and decreases in quantities of genes at every stage of training. Nevertheless, much higher levels of DEGs were observed in the blood, both upregulated and downregulated (Figure 2).

**Figure 3:**
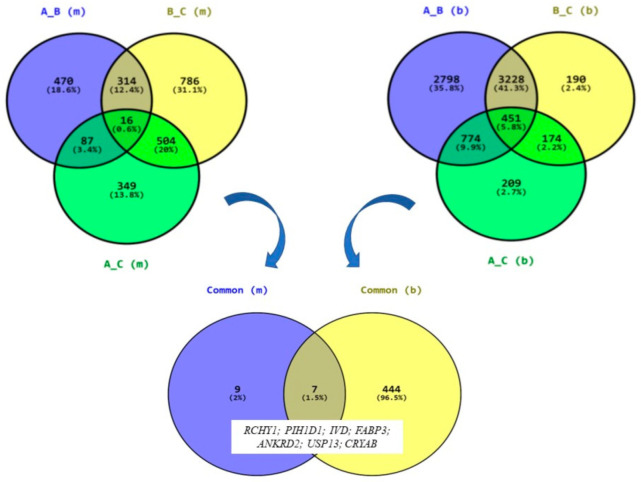
Venn diagrams illustrating the distribution of DEGs among three groups (A_B, B_C, and A_C) for muscles (m) and blood (b). The numbers inside the circles represent the count and percentage of elements within each category. The bottom Venn diagram shows the overlap between the two tissues, highlighting seven common elements: *RCHY1*, *PIH1D1*, *IVD*, *FABP3*, *ANKRD2*, *USP13*, and *CRYAB*.

Comparison of common genes in both tissues and periods shown seven common genes: *RCHY1* (ring-finger and CHY zinc-finger domain-containing 1), *PIH1D1* (PIH1 domain-containing 1), *IVD* (isovaleryl-CoA dehydrogenase), *FABP3* (fatty acid-binding protein 3), *ANKRD2* (ankyrin repeat domain 2), *USP13* (ubiquitin-specific peptidase 13), and *CRYAB* (crystallin alpha B). These genes are associated with fundamental processes, crucial for fitness and performance such as ubiquitination and deubiquitination (*USP13*), apoptosis (*PIH1D1*), intracellular metabolism and transport of long-chain fatty acids (*FABP3*), and muscle stress response, both oxidative and heat (*ANKRD2*, *USP13*, and *CRYAB*) (Figure 3).

## 4. Discussion

During domestication, human-driven selection pressures have resulted in the creation of highly specialized equine athletes distinctly separated by performance fields. These differences in phenotypes, including body shape, muscularity, body conformation, and responses to various types of exercise, are reflected in the genotype and are valuable models in terms of sports biology.

Whole-blood and muscle transcriptome profiling provides insights into the system-wide expression and abundance of all transcripts, highlighting the crucial role of muscle function in overall body functioning. Therefore, we utilized muscle and blood transcriptome profiling data to identify potentially shared transcripts. Our analysis identified only seven genes common across all sampling points and both tissues, which, due to their functions, have the potential for further exploration in the context of identifying molecular markers useful for monitoring the development of physical form in athletes. Arabian horses, due to their geographical origin and specific physiological adaptations, are used for long-distance endurance [26]. This type of sports specialization requires intense aerobic metabolism and maintenance of body heat and homeostasis [13], as well as muscle rearrangement toward slow-twitch muscle fibers capable of generating force during prolonged, steady, and intense exertion [27].

Our comprehensive methodological approach, with the use of information about genes differentially expressed in two tissues at the same time point, showed the *ANKRD2* (ankyrin repeat domain 2) gene encoding stretch-induced protein, mostly expressed in slow type 1 muscle fibers, is also downregulated in blood. The *ANKRD2* gene is a titin-binding protein, that plays a role in slow muscle function but is not involved in hypertrophy. Its activation is linked to the stress induced by the mechanosensing of stretch (load) and cellular ROS (reactive oxygen species). It plays a role in proliferation and apoptosis during myogenic differentiation. However, *ANKRD2* expression is positively correlated with physical exercise and acts as a guardian in response to AKT2-mediated phosphorylation induced by hydrogen peroxide in response to situations where there is risk of tissue damage due to inflammation [28]. Additionally its altered mRNA expression and protein level might contribute to muscle phenotype [29].

One of the crucial processes during exercise is skeletal muscle remodeling, driven by protein turnover, where ubiquitination (UB) and deubiquitination (DUB) act as key signaling messengers [30]. UB is a post-translational modification in which ubiquitin is attached to a target protein. This three-step process involves three enzymes: ubiquitin-activating enzyme (E1) in an ATP-dependent manner, ubiquitin-conjugating enzyme (E2), and ubiquitin-protein ligase (E3). Recently, there has been growing evidence of the role of protein ubiquitylation in skeletal muscle following exercise, particularly in removing damaged myofibrillar proteins [31], stabilizing sarcomeric integrity [32], and regulating myogenesis and insulin signaling as non-degradative signaling events [33]. Both the UB and DUB pathways, as revealed by our study, share common tissue-specific genes. For instance, the *USP13* gene can regulate energy metabolism through its interaction with the *ACLY* and *OGDH* genes, both of which encode enzymes necessary for the operation of the tricarboxylic acid cycle. *USP13* is also implicated in the modulation of autophagy via the deubiquitination of Beclin-1 [34], and adjusts proteins involved in the DNA damage response through DUB. Together with gp78, *USP13* maintains the balance between UB and DUB, thereby controlling the ER-associated degradation (ERAD) process [35]. Similarly, the *RCHY1* gene, also known as *PIRH2*, encodes an E3 ligase that regulates DNA damage through the ubiquitination of p53, Chk2, p73, and PolH [36]. Since skeletal muscle serves as the body’s largest protein reservoir, the mammalian/mechanistic target of rapamycin (mTOR), whether in complex 1 or complex 2, plays a central role in shaping muscle phenotype and influencing the health of distant tissues. Our investigation showed that whole blood and muscle share the *PIH1D1* gene transcript which is a defining component of the R2TP complex. The R2PTP complex includes Rvb1 and Rvb2, which are highly conserved, and the closely related AAA+ ATPases, RPAP3, and PIH1D1. This complex is involved in box C/D snoRNP and PIKK assembly with Hsp90, [37], and was to be found to be involved in apoptosis, where depletion of *PIH1D1* promotes apoptosis and caspase-3 activation [38]. Furthermore, *PIH1D1* regulates mTORC1 activity and assembly and positively regulates transcription of rRNA genes [39].

During prolonged endurance exercise, branched-chain amino acids (BCAAs), particularly leucine, undergo increased oxidation and promote skeletal muscle protein synthesis [40,41]. Leucine is recognized as a crucial amino acid, with its anabolic effects mediated through the activation of mTORC1. Deregulation of the IVD gene, which encodes isovaleryl-CoA dehydrogenase, in both blood and muscle, suggests intense BCAA catabolism, leading to the production of acetoacetate and acetyl-CoA. Endurance exercise promotes BCAA catabolism through fatty acid oxidation, as reflected in our results. The increased BCAA catabolism may serve to replenish the glutamate and ATP pools for glutamine synthesis, thereby facilitating ammonia detoxification post-exercise [42,43].

The Arabian horse is an exceptional animal model for investigating the impact of endurance on the organism. It has been shown to excel as an athlete, utilizing free fatty acids as a primary energy source rather than carbohydrates [44]. This hypothesis also supports the identified occurrence of *FABP3* deregulation in analyzed tissues. The *FABP3* gene, which encodes fatty acid-binding protein 3, is highly expressed in the heart and skeletal muscle and is one of the important regulators of lipid solubility, mobility, and utilization of fatty acids [45]. Additionally, it stimulates glucose uptake via AMPK-dependent *AS160* phosphorylation in skeletal muscle [46]. An increased level of *FABP3* mRNA is observed in endurance-trained athletes, indicating mobilization of fatty acids from extracellular and intracellular stores [47] and it might act as a biomarker of muscle injury during repeated exercise bouts [48]. The last gene deregulated by exercise in this study belonged to the mammalian lens alpha crystallin family. *CRYAB* is a heat shock protein that prevents protein aggregation and regulates homeostasis in muscles by interacting with the N- and C-terminal regions of Argonaute2. Interestingly, in *CRYAB* knockout mice, disturbances have been observed in the hypertrophy–atrophy signaling axis, along with impaired ability of satellite cells to regenerate skeletal muscle [49]. Furthermore, it acts as an immediate stabilizing cellular response following mechanical stress in skeletal muscle, with growing evidence indicating its potential role as a marker for the impact of resistance exercise loading on skeletal muscle fibers [50,51].

In recent years, several papers have been published on the genetics of racing performance in Arabian horses, providing valuable insights into the genetic basis of endurance and speed in these horses. However, these studies lack a more integrative perspective, regarding the interaction between different physiological systems.

Previous studies have shown that Arabian horses predominantly have type I muscle fibers, which favor fatty acid metabolism and enhance endurance capacity. The findings also indicate the presence of specific selection signatures, including genes associated with energy metabolism and adaptation to physical exertion. Transcriptomic analyses have allowed the identification of metabolic pathways involved in the response to long-term training and the effect of physical effort on bone homeostasis. Integrating these findings with research on muscle adaptation could provide a more comprehensive understanding of the mechanisms influencing Arabian horses’ performance.

## 5. Conclusions

Our results highlight the value of analyzing transcriptomic profiles across multiple tissues to gain insights into molecular responses to exercise. While the identified seven genes that overlap expression in two tissue types show potential, further research is needed to determine their relevance as potential biomarkers for training adaptation and performance assessment in endurance horses. This study contributes to the broader field of equine sports genetics and may serve as a foundation for future investigations, including potential applications in human athletics.

## Data Availability

The data presented in this study are openly available in: https://www.ncbi.nlm.nih.gov/geo/query/acc.cgi?acc=GSE88951 (accessed on 24 March 2024), https://www.ncbi.nlm.nih.gov/geo/query/acc.cgi?acc=GSE83404 (accessed on 24 March 2024).
